# Functional Liver Imaging in Radiotherapy for Liver Cancer: A Systematic Review and Meta-Analysis

**DOI:** 10.3389/fonc.2022.898435

**Published:** 2022-06-17

**Authors:** Pi-Xiao Zhou, Ying Zhang, Quan-Bin Zhang, Guo-Qian Zhang, Hui Yu, Shu-Xu Zhang

**Affiliations:** Radiotherapy Center, Affiliated Cancer Hospital and Institute of Guangzhou Medical University, Guangzhou, China

**Keywords:** liver neoplasms, functional liver imaging, radiotherapy, dose-response, radiation-induced liver toxicity (RILT)

## Abstract

**Backgrounds:**

Functional liver imaging can identify functional liver distribution heterogeneity and integrate it into radiotherapy planning. The feasibility and clinical benefit of functional liver-sparing radiotherapy planning are currently unknown.

**Methods:**

A comprehensive search of several primary databases was performed to identify studies that met the inclusion criteria. The primary objective of this study was to evaluate the dosimetric and clinical benefits of functional liver-sparing planning radiotherapy. Secondary objectives were to assess the ability of functional imaging to predict the risk of radiation-induced liver toxicity (RILT), and the dose-response relationship after radiotherapy.

**Results:**

A total of 20 publications were enrolled in descriptive tables and meta-analysis. The meta-analysis found that mean functional liver dose (f-MLD) was reduced by 1.0 Gy [95%CI: (-0.13, 2.13)], standard mean differences (SMD) of functional liver volume receiving ≥20 Gy (fV_20_) decreased by 0.25 [95%CI: (-0.14, 0.65)] when planning was optimized to sparing functional liver (P >0.05). Seven clinical prospective studies reported functional liver-sparing planning-guided radiotherapy leads to a low incidence of RILD, and the single rate meta-analysis showed that the RILD (defined as CTP score increase ≥2) incidence was 0.04 [95%CI: (0.00, 0.11), P <0.05]. Four studies showed that functional liver imaging had a higher value to predict RILT than conventional anatomical CT. Four studies established dose-response relationships in functional liver imaging after radiotherapy.

**Conclusion:**

Although functional imaging modalities and definitions are heterogeneous between studies, but incorporation into radiotherapy procedures for liver cancer patients may provide clinical benefits. Further validation in randomized clinical trials will be required in the future.

## Introduction

Hepatocellular carcinoma (HCC) is the fifth most common cancer and the third most common cause of cancer-related death globally, and its incidence is increasing year by year (1, 2). Moreover, the liver is the most common site of metastasis for other primary cancers (3). The liver was considered a contraindication to radiotherapy in the past because the radiation dose could not be safely delivered to the whole liver and could lead to acute radiation-induced liver toxicity (RILT) and even death (1, 4). Although, stereotactic body radiotherapy (SBRT) can provide a highly conformal dose of intense radiation to tumors while minimizing damage to organs at risk (OARs) and has become an effective treatment for liver tumors with excellent local control rates of 80% to 90% (5). However, RILT was a complex condition with a wide range of clinical symptoms ranging from an asymptomatic elevation of liver enzymes to liver failure and death, with an G3+ incidence of 5% to 36% in SBRT patients, limiting the implementation of high-dose radiotherapy (4–7). This risk was further increased by several pre-existing factors in the liver parenchyma, including hepatitis B virus and hepatitis C virus infection and cirrhosis (5). Until now, there was no specific treatment for RILT (4).

HCC patients have significant heterogeneity in liver function, which may be the result of organ structure, disease, or previous treatment injuries (8). The clinical liver function assessment was usually graded using Child-Turcotte-Pugh (CTP) tools, but it can be influenced easily by clinician’s subjective and other confusing factors (9). The further problem was that the widely used dose-volume constraint of the normal liver relies on anatomical CT imaging (which provides morphological information) and fails to consider liver function inhomogeneities in planning (5, 9).

Currently, functional liver imaging modalities include dual-energy CT, Magnetic Resonance Imaging (MRI), Single-Photon Emission Computed Tomography (SPECT), and Positron Emission Tomography (PET), all of which can provide quantitative visualization of liver function distribution. Integrate liver function 3-dimensional distribution information into planning for optimization (such as changing beams direction) to safely deliver a higher dose and minimize the ‘best functional’ (i.e., functional liver) liver dose (8, 10). Functional liver imaging complements anatomical CT imaging and provides insight into RILT beyond the existing anatomy-based dose-volume predictive model. Though functional imaging studies have shown a higher value in predicting radiation-induced lung injury than anatomical CT planning parameters (e.g., functional lung mean dose greater than lung mean dose), but its value in predicting RILT was unknown (11). In addition, there was no clear consensus on functional liver imaging modalities, functional liver definition, and functional planning optimization. Therefore, this systematic review was focused on evaluating the potential utility value of functional liver imaging in liver cancer radiotherapy.

## Materials and Methods

The systematic review was performed using structured search terms following the PRISMA guidelines (12). Our research questions regarding patients, interventions, comparisons, outcomes, and study design (PICOS) methods are described in **Supplemental A (Table 1)**. This systematic review and meta-analysis had been pre-registered on the PROSPERO (CRD42021257779).

### Search Strategy

We performed a systematic literature search in five electronic databases on April 10, 2021: PubMed, Embase, Cochrane, Sinomed, Chinese National Knowledge Infrastructure (CNKI). All eligible literature with a publication date between 1990 and search date was included. In PubMed and Embase databases, the search strategies of combining subject headings and free text words were adopted. The following subject headings searches were used: “Liver Neoplasms” AND “Radiotherapy”. All the ‘Entry Terms’ of the subject headings were used as free text words. These were finally combined with key words “functional liver”, “liver function”, or “functioning liver”. Searches in Embase database adopted a similar principle and were adjusted according to the database’s thesaurus. While in the Cochrane database, subject headings combined with keywords were used. The keywords search strategy was used in two Chinese databases. A manual secondary search of the reference list uncovered an additional 18 studies. There were no restrictions on language. The complete search strategy for each database was available in **Supplemental B**.

### Study Selection

The following study inclusion criteria were followed: (i) Functional liver imaging utilized in external radiotherapy for patients with liver cancer; (ii) Comparison of the differences in dose-volume parameters (DVP) between the functional liver sparing and the anatomical CT radiotherapy planning; or (iii) Investigate the ability or parameters of functional liver imaging to predict RILD (radiation-induced liver disease, defined as CTP score increase ≥2), and the dose-response relationship; or (iv) Exploring liver cancer patients’ RILD rate after delivery of functional liver protection planning-guided radiotherapy.

Editorials, letters, reviews, and case reports were excluded. When several publications on the same topic existed simultaneously from the same research team, the article with the wealthiest data or the latest publication was chosen. The number of patients included in each study was not less than five. Mean ± standard deviation (SD) or median (range) of the planning parameters that cannot be obtained were excluded from the meta-analysis.

### Data Collection

Data were extracted independently from each article by two reviewers and recorded in a prepared data collection form. Any differences between the extracted data from the two were resolved by negotiation or by a third reviewer.

Data collected included: first author, year of publication, study type, functional liver imaging modality, patient characteristics, number of patients, the definition of the functional liver, functional liver imaging technology parameters, information of radiotherapy planning, assessment of the dose-response relationship, the association between the functional liver dosimetric and clinical outcomes (RILT, prognosis). The dosimetric (fV_20_: functional liver volume receiving ≥20 Gy; f-MLD: mean functional liver dose) were collected from comparative studies of functional liver sparing and anatomical CT planning. Two reviewers evaluated literature (non-randomized controlled trials) quality using the Newcastle–Ottawa scale (NOS), and a score of seven or higher was considered a high-quality study. Differences were resolved through negotiation or by a third reviewer.

### Meta-Analysis

Meta-analysis was performed for the differences in f-MLD and fV_20_ between conventional anatomical and functional planning. Not all studies provide mean ± SD, and if raw data are not available, but median values and confidence intervals are provided, then the method described can be used to calculate (13). Some studies only provided the mean value without SD or only the difference between anatomical and functional planning, so these studies were also excluded.

Meta-analysis and corresponding plots were performed using the statistical software - Review Manager (RevMan) Version 5.4 (The Cochrane Collaboration, 2020) or Stata 16.0. Heterogeneity was assessed using the I^2^ statistic (I^2^ >50%, showed significant heterogeneity). Random-effects models were more applicable to mitigate heterogeneity than fixed-effects models, so random-effect model was selected for meta-analysis. We evaluated publication bias using funnel plots and Egger’s test checked (when the number of studies included in meta-analysis was ≥ 10, P value < 0.05 considered significant). Sensitivity analysis was performed by exclude studies one-by-one.

## Results

**Figure 1:**
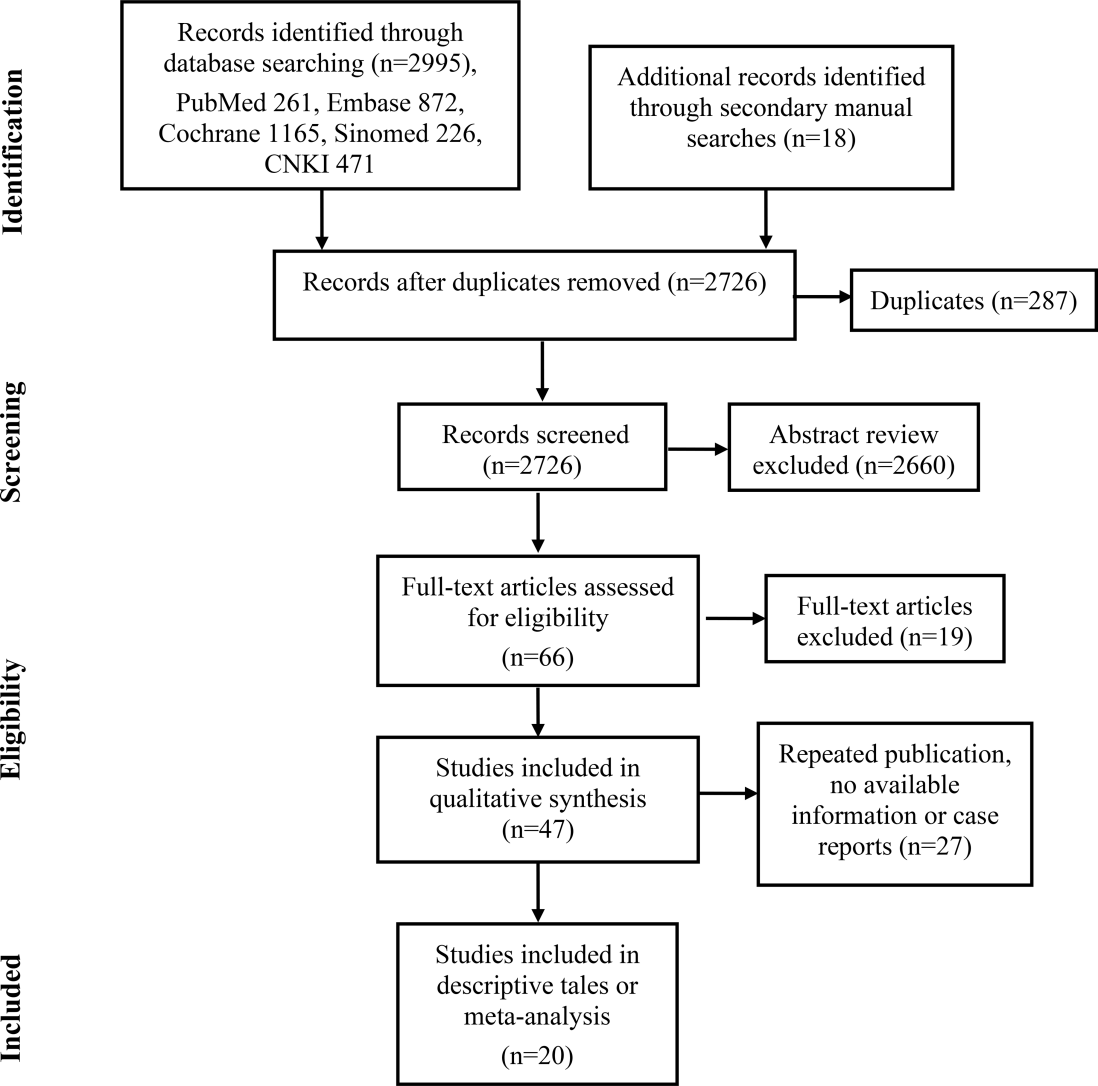
The flow diagram illustrates the screening and evaluation process (adapted from the PRISMA guidelines).

A total of 66 publications were enrolled in the full-text assessments. Forty-seven publications met the inclusion criteria for descriptive analysis (**Figure 1**). Twenty-seven of them were excluded because they were case reports, number of patients less than five, had no available information or had already included the latest or most informative publication of the same research team (14–40). Finally, the remaining twenty articles met the meta-analysis or descriptive tables (3, 5, 6, 9, 10, 41–55).

Nine functional liver imaging studies met inclusion criteria for comparison of functional liver and conventional anatomical planning dosimetric (3, 5, 41–44, 49, 50, 55) (**Table 1**). Almost all studies showed statistically significant decrease of functional liver dose in the functional liver-sparing planning compared to the conventional anatomical planning, without significant change in the target consistency index (CI), homogeneity index (HI), or OARs dose. Some studies reported that f-MLD and fV_20_ were associated with the collected data endpoints (RILD), and had sufficient published data available for meta-analysis. According to the NOS score, all studies were of high quality, and the evaluation details are shown in **Supplemental A (Table 2)**.

**Table 1:** Provides detailed descriptions of each study, including patients’ information, functional imaging/planning technique utilized, functional liver definition and key findings.

Reference	Patients	ReferenceTypes	Characteristics	Technology,contrast agent,RT technique	Definition FL	Benefit of FL sparing (% difference between means)	Comparing planning quality
Ohira et al. (2020) (3)	10 (15)	Article	HCC 86% GTV: NS 36.8-65Gy	DECT (Iodine) VMAT (SBRT)	NID<0.46	*f-MLD ↓ 1.9 Gy *fV5 ↓ 58.7 cc, 53.8 cc, 27.2 cc, 13.3 cc	No SS change CI, SS HI ↑; No SS change to OARs
Toya et al. (2019) (5)	11	Article	HCC 100% PTV: 42.9 cm3 (M) 50Gy	SPECT/CT (99mTc-GSA) VMAT (SBRT)	60%-80% of max	*fV5-20 ↓ 1.5%, 2.1%, 1.4%, 0.7%	No SS difference CI, HI; No SS change to OARs
Furukawa et al. (2020) (41)	10	Article	HCC 100% GTV: NS 40-50Gy	SPECT (^99m^Tc-HIDA) SBRT	25%-100% max; 50%-100% max	*f-MLD↓ 2Gy/3Gy; *fV_D<15Gy_ ↓ 50%/41.9%	No SS difference CI; Dose to OARs NS
Tsegmed et al. (2017) (42)	20	Article	HCC 100% PTV: 16.2 cm3 48Gy	MRI (Gd-EOB) IMRT (SBRT)	HBP L/S ≥1.5	*f-MLD ↓ 0.5 Gy *fV5-20 ↓ 3%, 3%, 1.9%, 0.7%	SS CI ↑; No SS change to OARs
Bowen et al. (2015) (43)	10	Article	HCC 100% GTV: (M) 88 cm^3^ 37.5-60Gy	SPECT/CT (^99m^Tc-Sc) VMAT/PBS	43%-90% of L/S max	*f-MLD ↓ 20%	Dose to PTV or OARs NS
Long et al. (2018) (44)	17	Article	HCC: 100% GTV: 29.5cc 27.5-50Gy	SPECT (^99m^Tc-HIDA) SBRT	50%-100% of max	*f-MLD ↓ 1.18 Gy *fV_D<15Gy_ ↑ 0.15%	No SS difference CI; Dose to OARs NS
Simeth et al. (2018) (49)	10	Conference	HCC 100% GTV: NS 55Gy	MRI (Gd-EOB) -	36% to max	*f-MLD ↓ 10.5%	Dose to PTV or OARs NS
Fode et al. (2017) (50)	7	Article	6 mCRC/1 IHC CTV: 25.1cc (M) 45-56.25Gy	PET/CT(^18^F-FDG) VMAT (SBRT)	10%/20%/30% volume with the highest SUV	*f-MLD↓ 0.8/0.6/0.4 Gy; fV_D<15Gy_ ↑ 6%/4%/3%	No SS difference CI; Dose to OARs NS
Lin et al. (2019) (55)	10	Article	HCC 100% PTV: 122.7 cm3 50-62Gy	MRI (Gd-EOB) IMRT	T1WI, high signal area in HBP (20min)	*f-MLD ↓ 0.54 Gy *fV5-20 ↓ 0.41cm^3^,0.32cm^3^, 0.22 cm^3^, 0.14 cm^3^	No SS difference CI and HI; No SS change to OARs

Key: *, denotes statistically significant result; No., number; FL, functional liver; mCRC, metastatic colorectal cancer; IHC, intrahepatic cholangiocarcinoma; ^18^F-FDG, 2-[18F] fluoro-2-deoxy-D-galactose; SUV, standard uptake values; f-MLD, functional liver volume mean dose; fV_D<15Gy_, volume of functional liver receiving less than 15 Gy; SS, statistically significant; CI, conformity index; OARs, organs at risk; NS, non-specified; HCC, hepatocellular carcinoma; ^99m^Tc-HIDA, technetium-99-mebrofenin (Tc99m) hepatobiliary iminodiacetic acid (HIDA); ^99m^Tc-Sc, ^99m^Tc-Sulphur colloid; PBS, proton pencil beam scanning; L/S, liver-to-spleen ratio; DECT, Dual-energy computed tomography; NID, normalized iodine density; fVx, functional liver volume receiving ≥x Gy; HI, homogeneity index; MU, monitor unit; SC, spinal cord; PTV, planning target volume; Gd-EOB-DTPA, Gadolinium ethoxybenzyl diethylenetriamine pentaacetic acid; HBP, hepatobiliary phase; T1WI, T1 weighted image. ↑, increase; ↓, decrease.

**Figure 2:**
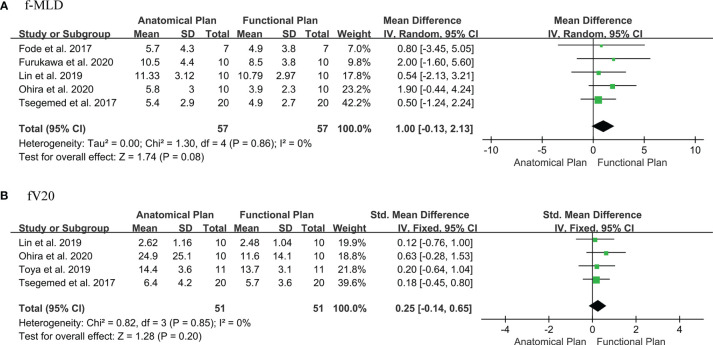
Forest plot of the difference in dose-volume parameters of functional liver between the functional liver-sparing and conventional anatomical plans. **(A)** f-MLD (functional liver mean dose); **(B)** fV_20_ (functional liver volume receiving ≥ 20 Gy).

Eight publications were selected for f-MLD meta-analysis (3, 41–44, 49, 50, 56). Two articles used different thresholds to defined functional liver, and compared dose differences between functional liver-sparing and conventional anatomical planning (41, 50). Some studies were not included in the meta-analysis because they were incomplete (only mean value or percentage difference), and raw data were unavailable (44, 49, 53). When only one threshold was included from each study (the threshold which showed the maximum difference between conventional and functional liver-sparing planning), the f-MLD was decreased by: 1.0 Gy [95%CI: (-0.13, 2.13), I^2^ = 0%] between functional liver-sparing and conventional anatomical planning (**Figure 2A**). Sensitivity analysis found the result was robust, and no significant publication bias was identified by visual observation from the funnel plot (**Supplemental A Figure 1**). Four papers were eligible for fV_20_ meta-analysis (3, 5, 42, 55). The SMD was used because the fV_20_ value in these four studies were expressed in different forms (cc, cm^3^ and percentage). The fV_20_ SMD was decreased by: [0.25, 95%CI:(-0.14, 0.65), I^2 =^ 0%] between functional liver-sparing and conventional anatomical planning (**Figure 2B**). Currently, there were significant statistical difference in f-MLD and fV_20_ between functional liver-sparing and conventional anatomical planning (P >0.05).

**Figure 3:**
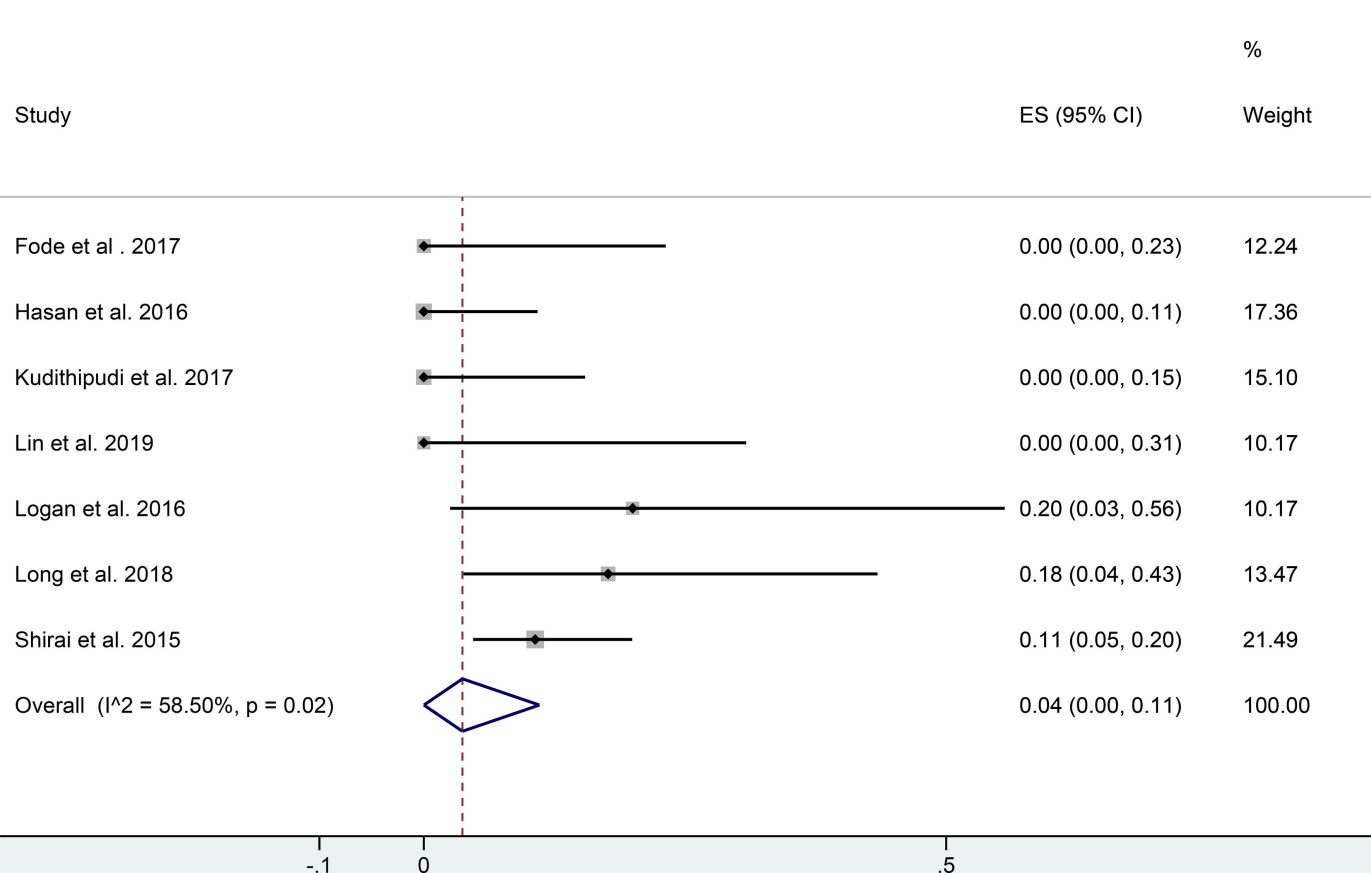
Forest plot of single rate mete-analysis in incidence of RILD for functional liver protection planning guided radiotherapy. (RILD, radiation-induced liver disease).

Four of the studies showed that functional liver imaging was statistically significant in predicting the endpoint of RILT, while no correlation was found with anatomic planning (9, 44, 53, 54) (**Table 2**). Although the endpoints used to evaluate RILD were inconsistent across studies. Seven studies evaluated RILT after functional liver-sparing planning-guided radiotherapy, six of which were prospective and one retrospective (6, 44, 48, 51–53, 55) (**Table 2B**). A total of 180 patients were included, 111 had a baseline CTP score A, and the rest had score B or higher (55 patients), and one publication did not describe baseline liver function. Altogether, thirteen participants were described to have developed RILD. Logan et al. (48) showed that patients who developed RILD had poorer baseline liver function (both CTP B/C). The single rate meta-analysis of the incidence of RILD in the included studies showed an incidence of 0.04 [95%CI: (0.00, 0.11), I^2^ = 58.5%, P <0.05] when functional liver-sparing planning-guided radiotherapy (after double-arcsine transformations) (**Figure 3**). And three studies described correlations between functional liver imaging and prognosis, two of which demonstrated the functional liver dose-volume parameters to predict prognosis (9, 47).

**Table 2:** Description of functional liver imaging correlating with clinical liver function, radiation-induced liver disease and prognosis.

a) Conventional anatomical planning radiotherapy						
Reference	Patients	ReferenceType	Characteristics	Imaging type, Plan technique, Definition FL	Morbidity or correlation with CTP (RILD) after RT	Prognosis
Schaub et al. (2018) (9)	47	–	HCC: 95.7% GTV: 33.43cm^3^ (M) CTP: 29A 18B/C	SPECT/CT (99mTc-Sc) SBRT/PRT >30% of max	11 RILD (CTP +2) TLF and L/Smean (*CTP +2).	f-MLD/fV20 (*RILD-special survival, AUC 0.74/0.78, cutoff 23Gy/36%)
Bowen et al. (2016) (47)	30	–	HCC 100% GTV: 15 cm^3^ (M) CTP 16A, 12B, 2C	SPECT/CT (^99m^Tc-Sc) - 20%-70 % of max	–	TLF (cutoff >0.30) (*OS)
Nakamura et al. (2015) (54)	30	–	HCC 100% CTP 26A, 4B	MRI (Gd-EOB) SBRT	W-LSC (*CTP +2, AUC 0.83, cutoff 1.88)	–
b) Functional liver protection planning-guided radiotherapy						
Reference	Patients	Reference Type	Characteristics	Imaging type, Plan technique, Definition FL	Morbidity or correlation with CTP (RILD) after RT	prognosis
Fode et al. (2017) (7)	14 (15)	PO	mCRC 100% -	PET/CT (^18^F-FDG) IMRT (SBRT) 10%-30% volume with highest SUV	No G3+ acute morbidity (No RILD)	Last follow-up (M 16.6 mo), 10 survived
Long et al. (2018) (44)	17	PO	HCC: 100% GTV: 29.5cc CTP: 12A 5B	SPECT (^99m^Tc-HIDA) SBRT 50%-100% max	3 RILD (CTP +2, 6 mo); 10 patients developed decompensation (*fV_D<15Gy_, AUC 0.929, cutoff < 2.915%/min/m^2^)	–
Logan et al. (2016) (48)	10	PO	HCC 100% - CTP: 5A, 5B	SPECT/CT (^99m^Tc-Sc) IMRT/PRT 20% to 50% of max	2 RILD (both CTP B8-9)	OS (med) was 116 days; FLV (*OS, SPECT thresholds of 30%, 35%, 40%, 43%, and 45% of max)
Kudithipudi et al. (2017) (51)	22 (39)	PO	HCC 100% PTV: 293.0cm^3^(M) CTP: 14A, 8B	SPECT (^99m^Tc-Sc) SBRT/FSRT Related body surface area	No RILD CTP score preservation rate 59% (1 year)	OS: 59% (2 years)
Hasan et al. (2016) (52)	32	PO	HCC 100% CTP: 32A	SPECT (^99m^Tc-Sc) -	No RILD 73%/56% retained CTP A (1/2 years)	OS: 87%/63% (1/2 years)
Shirai et al. (2015) (53)	75	RO	HCC with PVTT GTV: 448.7cm^3^ (M) CTP: 39A 36B/C	SPECT (^99m^Tc-GSA) 3D-CRT Areas of uptake exceeds tumor	8 RILD (CTP +2) fV20 (*CTP +1 vs. +2, AUC 0.792, cutoff 26.4%)	1 year, 2 years, 5 years OS were 47.0%, 20.4%, 11.2%;
Lin et al. (2019) (55)	10	PO	HCC 100% PTV: 122.7 cm^3^ (M) CTP: 9A 1B	MRI (Gd-EOB) IMRT T1, high signal area in HBP (20min)	No RILD	–

Key, *, functional liver imaging more predictive risk of CTP +1/RILD (CTP +2) or prognosis; PO, prospective; RO, retrospective; FL(V), function liver (volume); M, mean; Med, median; mo, months; CTP, Child-Turcotte-Pugh; OS, overall survival; SS, statistically significantly; +2, score increase ≥2; G2+, grade ≥2+; HCC, hepatocellular carcinoma; GTV, gross target volume; AUC, area under the curve; NS, non-specified; mCRC, metastatic colorectal cancer; ^18^F-FDG, 2-[18F] fluoro-2-deoxy-D-galactose; SUV, standard uptake values; ^99m^Tc-Sc, ^99m^Tc sulfur colloid; TLF, FLV×L/S_mean_; L/S_mean_, liver-to-spleen uptake ratio; LM, liver metastases; f-MLD, functional liver volume mean dose; BBT, broad biochemical toxicity (defined as a 50% increase in each of the 3 measured liver enzymes); PRT, proton radiotherapy; ^99m^Tc-HIDA, technetium-99-mebrofenin (Tc99m) hepatobiliary iminodiacetic acid (HIDA); fV_D<15Gy_, volume of functional liver receiving less than 15 Gy; fV20, functional liver volume receiving ≥20Gy; PVTT, portal vein tumor thrombus; ^99m^Tc-GSA, Tc-99 m-galactosyl human serum albumin; Gd-EOB-DTPA, Gadolinium ethoxybenzyl diethylenetriamine pentaacetic acid; HBP, hepatobiliary phase; W-LSC, weighted liver-spleen ratio;

Four publications described the dose-response relationship between radiation dose received and liver function decline (6, 10, 45, 46) (**Table 3**). Three studies used linear models (6, 10, 46), and another study had a sigmoid-shaped dose-response curve. Changes in liver function with dose were similar across the four studies, despite differences in population heterogeneity, radiotherapy techniques, prescription dose, time points, and dose-response model parameters.

**Table 3:** Description of dose–response relationship after radiotherapy.

Reference	Study Type	No.	Characteristic	Planning technique	Imaging Type,contrast agent	Time points	Dose–response model and parameters
Fode et al. (2017) (6)	PO	14	Age: (M) 72 LM 100% -	IMRT (SBRT) 45-60Gy	PET/CT ^18^F-FDG:100 (MBq)	1 month	**linear model** approximately -1.2% metabolic function per Gy, D_50_ of 22.9 Gy/3 F
Wang et al. (2013) (10)	PO	14	Age: 44-83 10 HCC, 3 CC, 1LM -	SBRT (8) 33Gy IMRT (3) 52Gy 3D-CRT 62Gy	SPECT ^99m^Tc-HIDA (10 mCi)	1 month	**linear model** approximately -0.33% of HEF per Gy
Price et al. (2018) (45)	PO	15	- HCC 100%	SBRT/PRT NA	SPECT/CT ^99m^Tc-Sc (-)	1 month	**Gompertz model** Maximum uptake median -0.11% per Gy, D_50_ 13Gy.
De Bari et al. (2018) (46)	PO	6	Age: (M) 69 3 LM and 3 HCC PTV: 130 cc	SBRT 30 Gy	SPECT/CT ^99m^Tc-HIDA (200MBq)	3 months	**linear model** mean -0.78% of perfusion per Gy

Key, PO, prospective observational; M, mean; LM, liver metastases; HCC, hepatocellular carcinoma; ^99m^Tc-HIDA, technetium-99-mebrofenin (Tc99m) hepatobiliary iminodiacetic acid (HIDA); CC, cholangiocarcinoma; HEF, hepatic extraction fraction (obtained from SPECT data); ^18^F-FDG, 2-[18F] fluoro-2-deoxy-D-galactose. ^99m^Tc-Sc, ^99m^Tc-Sulphur colloid; PRT, proton radiotherapy.

The most frequently utilized functional liver imaging technique in included literature was SPECT (tracer: ^99m^Tc-Sc, ^99m^Tc- HIDA or ^99m^Tc-GSA) with 14 publications, followed by MRI (Gd-EOB-DTPA) with four papers, PET (^18^F-FDG) with two studies, and dual-energy CT (Iodine) with one literature. Thirteen studies provided functional liver imaging scans parameters. Many studies used one or more methods to suppress the effect of breathing motion in both the accuracy of liver tumor volume measurements and image registration (e.g., end-exhale CT, four-dimensional CT, abdominal compression, breathing-hold, or Calypso-guide gating). Several registration systems or toolkits were mentioned in the literature. For instance, Smartadapt/MIM software, Velocity software, insight segmentation, and registration toolkit (**Supplemental A Table 3**).

## Discussion

The current evaluation of the liver radiotherapy planning was based on the assumption of uniform and consistent liver function. Although with the advancement of radiotherapy technology, the dose distribution in the target area has been improved, and radiation dose to surrounding OARs has been reduced. However, there are still a minority of liver cancer radiotherapy patients who will have a significant RILT, leading to treatment interruption or even death by acute liver failure. However, previous studies have shown that functional liver imaging can identify the distribution of liver function and could be integrate it into radiotherapy planning to protect the higher-functioning liver volumes. That is the first systematic review and meta-analysis of functional liver imaging in radiotherapy for liver cancer. Meta-analysis indicated that functional liver preservation radiotherapy planning could potentially reduce f-MLD and fV_20_, but no statistical significance. Because some studies were abandoned before meta-analysis due to incomplete data, so future functional liver-sparing comparative planning studies should clearly state the primary functional liver mean doses differences (± SD). Dose constraints regimens and parameters optimization settings also should be clearly provided for functional liver-sparing and conventional anatomical planning to guarantee consistency. However, the single rate meta-analysis of the incidence of RILD with functional liver-sparing planning-guided radiotherapy showed that the incidence of RILD was only 4%, which was lower than previously reported for the conventional anatomical planning radiotherapy.

The maximum clinical significance of functional liver imaging planning was to reduce the incidence of RILT without reducing overall survival. In the included literature, studies showed that functional liver dose-volume parameters (e.g., fV_20_) helped predict the occurrence of RILT. In clinical, it was a challenge to improve the survival rate, and reduce the incidence of RILT of HCC patients with first portal vein tumor thrombus (PVTT). However, Shirai et al. (18) showed that no RILD occurred in patients with large hepatocellular carcinoma (greater than 14 cm) or combined PVTT who received the functional liver-sparing planning-guided radiotherapy (38). In terms of prognosis, Shirai et al. (18) showed that median overall survival in HCC patients with PVTT treated with functional liver-sparing planning-guided radiotherapy was comparable to the previously reported PVTT radiotherapy combined with transcatheter arterial chemoembolization or surgical hepatectomy. At present, there is not enough data to establish a probability model of normal tissue complications. Future publications should provide sufficient information on the value of functional liver dosimetric and anatomical liver dosimetric to predict RILT, which can be used to guide the establishment of functional liver-based dose limitations.

A similar dose-response relationship was observed on the functional liver imaging after radiotherapy, which decreased with the increase of radiation dose. Most of the publications evaluated the time points for evaluation were about one month after RT treatment, and only one was evaluated at three months. The functional imaging modality used in all publications was nuclear medicine imaging. Currently, there was not enough data to assess the best functional liver imaging modalities.

The definition of the functional liver was inconsistent in included publications (e.g., for SPECT, functional liver was defined as the percentage of maximum liver radioactivity counts (ranging from 10%, 20%, 30%, 50% to 100% max), the ratio to heart or spleen radioactivity counts, etc.), and no clear clinical evidence to guide the optimal definition. Furukawa et al. (41) showed that functional liver defined as > 50% of max radioactive counts with f-MLD decreased beyond >25% of maximum in functional liver-sparing planning (in SPECT/CT). Theoretically speaking, it was best to avoid radiation doses to liver tissue other than the tumor, but this was impossible with current radiotherapy techniques. However, it was possible to establish non-overlapping functional liver regions by defining different thresholds so that the more functional liver tissue should give more weight to protection without sacrificing conformity of tumor target volume and other OARs.

In the systematic review, 20 studies utilized different functional imaging techniques and radioactive tracers (contrast agents), most of which used nuclear medicine imaging. SPECT provides three-dimensional imaging of indirect or direct liver function by injecting different radiotracers. SPECT combined with low-resolution CT can improve tissue contrast and the accuracy of local radioactivity uptake estimation (47). The radioactive tracer ^99m^Tc-HIDA is transported to hepatocytes *via* albumin to be taken up by organic anion transport protein (OATP1 B1/B3) and excreted in the biliary system without bio-metabolic conversion (55, 56). To exclude individual metabolic differences, divided by body surface area (liver uptake rate %/min/m2), De Graaf et al. (56) suggested the functional liver defined as 30% of the maximum radioactivity count. ^99m^Tc-GSA is binding specifically to the desialic acid glycoprotein receptor expressed on hepatocytes, and suggested defining voxels below 54% of the maximum radioactivity count as background (56). ^99m^Tc-Sc is taken up by Kupffer cells of the hepatic reticuloendothelial system, and its activity is closely related to liver function (43). PET/CT functional imaging provides better spatial and temporal resolution than SPECT (57). ^18^F-FDG is a radio-labeled galactose analogue that is metabolized by intrahepatic galactokinase and can be used to noninvasively quantify local hepatic metabolic function and visualize metabolic heterogeneity (50, 58). MRI-based functional imaging seamlessly connects to clinical examinations workflow, has a higher temporal and spatial resolution, and does not rely on ionizing radiation (59). Gd-EOB-DTPA, which is also selectively taken up by functional hepatocytes *via* OATP1 B1/B3, resulted in a significant shortening of T1 values in the functional liver. This leads to advancing the signal peak on T1WI to 20 min in the hepatobiliary phase in the functional liver (55, 60). DECT uses two different X-ray photon energies to quantitatively measure iodine density in tissues, which with high spatial resolution, high reproducibility, and low cost (3).

The modalities and acquisition parameters of functional liver images will affect the reconstruction and registration with anatomical CT images. Of the included publications, twelve described the image registration methods, most of which used MIM software, Smartadapt software, Velocity software, and insight segmentation and registration toolkit. The accuracy of image registration was critical in which the registration error between functional and anatomical CT images may lead to the spared inaccuracy of functional liver volume. Fukumitsu et al. (61) showed that for liver image registration, the performance of MIM and Velocity was generally similar. Insight segmentation and registration toolkit (ITK) was used to match image data, and the assessment results can be visualized and support different matching tasks in a clinical setting (62).

There were also some limitations in our study. First, published studies included in this review were in a small number of patients cohort and heterogeneous (patients characteristics, functional imaging modalities, radiotherapy techniques). It might be that functional liver imaging is still in the stage of clinical exploration and there are various treatment options available for liver cancer (such as early stage mostly choosing surgical resection, and local advanced stage opting for systemic therapy combined with radiofrequency ablation or intervention, etc.). Second, f-MLD and fV_20_ meta-analysis showed that both exhibited no statistically significant differences between functional liver preservation and anatomical plans (although all studies indicated a significant reduction). However, there was no significant heterogeneity or publication of bias. It might be associated with the number of patients, functional liver definition, and the difference of optimization. Negative results do provide a stronger warning to later investigators, reminding them to include larger study samples, report data in detailed (mean ± SD) or provide raw data in the appendix for data extraction, and give greater priority (weight) to functional liver in functional liver-sparing planning design. Third, most of the publications reviewed comprehensively did not report sufficient information to be included in the meta-analysis (e.g., no SD). And different studies reported inconsistent dose-volume parameters in functional plans (e.g., fV_15_, fV_<15Gy_), and inconsistent parameter units (such as cm^3^, cc, or percentage) compared with anatomical plans. Fourth, although the single rate meta-analysis of RILD incidence in the prospective observational studies of functional liver-sparing planning-guided radiotherapy indicated the potential to decrease RILD (but both SBRT and 3D-CRT were included, subgroup analysis was not performed). However, the reported endpoints of RILT were inconsistent (including CTP score increase 1 or 2 (RILD), or liver function decompensation), which may be a weakness of the current studies. In the future, sufficient randomized clinical intervention trials should be established to examine the differences in RILD incidence between functional liver-sparing and conventional anatomical planning. Probabilistic modeling of normal tissue complications should also be investigated to provide the optimal dose-limiting regimens for functional liver preservation planning-guided radiotherapy.

## Conclusion

The 20 studies in this systematic review suggested that functional liver imaging provided information on functional dose-volume parameters that can more acuate predict the risk of RILT than anatomical CT (e.g., fV_20_). We found a similar dose-response for functional liver imaging after radiotherapy, indicating the potential to integrate functional liver imaging into treatment planning to decrease functional dose metrics. The meta-analysis showed that there were insufficient data to confirm that functional liver-sparing planning significantly reduced f-MLD and fV_20_ compared with anatomical CT planning. Different studies have used a wide variety of functional liver thresholds, and no standard threshold has been established. Functional liver-sparing planning-guided radiotherapy could reduce the incidence of RILD, but has yet to be validated in prospective randomized clinical intervention trials.

## Data Availability Statement

The original contributions presented in the study are included in the article/**Supplementary Material**. Further inquiries can be directed to the corresponding author.

## Author Contributions

P-XZ performed the study concept and design, and wrote the manuscript. YZ and G-QZ performed literature collection, and data analysis. Q-BZ and HY conducted literature screen, data collection, and quality evaluation. S-XZ verified data and revised the manuscript. All authors contributed to the article and approved the submitted version.

## Conflict of Interest

The authors declare that the research was conducted in the absence of any commercial or financial relationships that could be construed as a potential conflict of interest.

## Publisher’s Note

All claims expressed in this article are solely those of the authors and do not necessarily represent those of their affiliated organizations, or those of the publisher, the editors and the reviewers. Any product that may be evaluated in this article, or claim that may be made by its manufacturer, is not guaranteed or endorsed by the publisher.
