# Supplementary material for: Functional Liver Imaging in Radiotherapy for Liver Cancer: A Systematic Review and Meta-Analysis
Source: Front Oncol. 2022 Jun 17;12:898435. doi: 10.3389/fonc.2022.898435 (PMC9247161; doi:10.3389/fonc.2022.898435)
Supplement: Supplementary file 1 [file DataSheet_1.docx]

Table 1. PICOS table for research question. fV20: functional liver volume receiving ≥20Gy; f-MLD: functional mean liver dose; OARs: organs at risk.

|  | Contents |
| --- | --- |
| Patients | Liver cancer patients (primary or metastatic liver cancer) |
| Intervention | Functional liver protection planning using functional liver imaging for radiotherapy |
| Comparison | Anatomical CT planning |
| Outcome | **Primary:** 1. Benefit of planning to functional liver compared to anatomical liver (dosimetric, planning quality and OARs);  2. Benefit of functional liver sparing planning-guided radiotherapy for liver cancer;  **Secondary:** 1. The ability of functional liver imaging to predict risk of radiation-induced liver disease (RILD) compared to anatomical liver CT; Functional liver dosimetric correlation with RILD.  2. Description of the dose-response relationship after radiotherapy functional liver imaging;  3. Description of the types of functional liver imaging, functional liver definition and radiotherapy planning. |
| Study | Retrospective or prospective study in humans |

Table 2. Results of quality assessment using the Newcastle-Ottawa Scale for case-control studies.

| Reference | Selection | | | | Comparability  Comparability of cases and controls on the basis of the design or analysis | Exposure | | | Score |
| --- | --- | --- | --- | --- | --- | --- | --- | --- | --- |
|  | Is the case definition adequate | Represe-ntativeness of the cases | Selection of Controls | Definition of Controls |  | Ascertain-ment of exposure | Same method of ascertainment for cases and controls | Non-Response Rate |  |
| Fode et al. (51)  2017 | - | ☆ | ☆ | ☆ | ☆☆ | ☆ | ☆ | ☆ | 8 |
| Furukawa et al. (42)  2020 | - | ☆ | ☆ | ☆ | ☆☆ | ☆ | ☆ | ☆ | 8 |
| Ohira et al.(3)  2020 | - | ☆ | ☆ | ☆ | ☆ | ☆ | ☆ | ☆ | 7 |
| Toya et al.(5)  2019 | - | ☆ | ☆ | ☆ | ☆ | ☆ | ☆ | ☆ | 7 |
| Tsegmed et al.  (43)  2017 | ☆ | ☆ | ☆ | ☆ | ☆ | ☆ | ☆ | ☆ | 8 |
| Lin et al.(56)  2019 | ☆ | ☆ | ☆ | ☆ | ☆ | ☆ | ☆ | ☆ | 8 |

Table 3. Details of functional liver image radiotherapy treatment in the included studies

| Reference | Imaging Type | Reduce breathing motion effects | RT technique | Registration tools | Scanning parameters |
| --- | --- | --- | --- | --- | --- |
| Fode et al. (51)  2017 | PET/CT | AC and 4DCT | VMAT (SBRT) | Smartadapt /MIM Software | FOV: 21.6 cm, (50 mAs, 120 kV, pitch 0.8, slice thickness 5 mm) |
| Fode et al. (6)  2017 | PET/CT | AC or Calypso-guide gating, 4DCT and breath-hold | IMRT (SBRT) | Smartadapt /MIM Software | FOV: 21.6 cm, slice thickness 3 mm, matrix 336 |
| De Bari et al. (47)  2018 | SPECT/CT | 4DCT, online fiducial-based  tracking | SBRT | Velocity software | 36 frames of 10 seconds, 128×128 matrix |
| Wang et al. (11)  2013 | SPECT/CT | - | 3DCRT /IMRT /  SBRT | - | FOV: 500×500×355 mm^3^; 128 ×128 matrix |
| Furukawa et al. (42)  2020 | SPECT | AC and 4DCT | SBRT | MIM Software | - |
| Bowen et al. (44)  2015 | SPECT/CT | breath-hold and end-exhale CT | VMAT /PRT | MIM Software | 64 views, 20 s/view, 180° arc |
| Bowen et al. (48)  2016 | SPECT/CT | breath-hold, end-exhale CT and AC | - | MIM Software | 64 views, 20 s/view, 180° arc |
| Ohira et al. (3)  2020 | DECT | 4D-DECT /breath-hold DECT | VMAT (SBRT) | - | 140/80 kVp, FOV: 500 mm, rotation time 0.5s; slice thickness 2.5 mm; |
| Toya et al. (5)  2019 | SPECT/CT | AC | VMAT (SBRT) | Velocity AI | 60 steps, 15 s/step, 360°, 128 × 128 matrix; |
| Price et al. (46)  2018 | SPECT/CT | breath-hold, 4DCT, end-exhale CT and AC | SBRT /PRT | MIM Software | SPECT reconstructed 4.54 mm; CT slice thickness 2.5 mm; |
| Schaub et al. (10)  2018 | SPECT/CT | Fiducial markers, breath-hold, 4DCT and AC | SBRT /PRT | MIM Software | - |
| Long et al. (45)  2018 | SPECT | - | SBRT | MIM Software | - |
| Tsegmed et al. (43)  2017 | MRI | breath-hold | IMRT (SBRT) | insight segmentation and registration toolkit | 3-mm thickness |
| Kudithipudi et al. (52)  2017 | SPECT | 4DCT | SBRT/FSRT | - | - |
| Shirai et al. (54)  2015 | SPECT | - | 3DCRT | - | 90 steps, 360°, 128 ×128 matrix |
| Lin et al. (56)  2019 | MRI | 4DCT | IMRT | - | T1WI: TR 235ms，TE 2.2ms; T2WI: TR 2000ms，TE 75ms; FOV: 328×350 mm，matrix 240×320 |
| Nakamura et al. (55)  2015 | MRI | breath-holding, end-exhale CT | SBRT | insight segmentation and registration toolkit | FOV: 36 cm, matrix 256×192; TR/TE 3.3/1.6 ms; 3-mm thickness; |

Key: FOV: field of view; 4DCT:4-dimensional computed tomography; TR: repeat time; TE: echo time; PRT: proton radiotherapy; FSRT: Fractionated Stereotactic Radiotherapy; AC: abdominal compression;


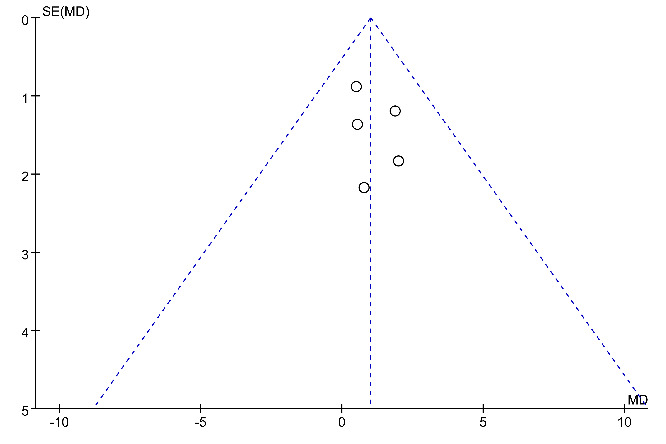


Fig. 1. Funnel plots of f-MLD meta-analysis (optimal threshold).
